# Experimental *Trypanosoma cruzi* Infection and Chagas Disease—A Word of Caution

**DOI:** 10.3390/microorganisms11061613

**Published:** 2023-06-19

**Authors:** André Talvani, Mauro Martins Teixeira

**Affiliations:** 1 Laboratório de Imunobiologia da Inflamação, Departamento de Ciências Biológicas, ICEB, Universidade Federal de Ouro Preto, Ouro Preto 35402-136, MG, Brazil; 2 Pós-Graduação em Saúde e Nutrição, Escola de Nutrição, Universidade Federal de Ouro Preto, Ouro Preto 35402-145, MG, Brazil; 3 Pós-Graduação em Ciências da Saúde: Infectologia e Medicina Tropical, Faculdade de Medicina, Universidade Federal de Minas Gerais, Belo Horizonte 30130-100, MG, Brazil; mmtex.ufmg@gmail.com; 4 Departamento de Bioquímica e Imunologia, ICB, Universidade Federal de Minas Gerais, Belo Horizonte 31270-901, MG, Brazil

The physician Carlos Chagas (1879–1934) described the protozoan parasite *Trypanosoma cruzi* and discovered a new illness named American trypanosomiases or Chagas disease (Chagas, 1909). In the last few decades, we have seen a plethora of new information about the biology of this parasite, the hematophagous vector (Triatomminae), the mammalian reservoirs, the screening of potential tripanocidal compounds and the pathological and clinical findings related to human cardiac, neurological and digestive diseases [1,2,3,4,5]. In addition, substantial new information on the molecular, therapeutic and immunological aspects of Chagas disease [6,7,8,9,10] and updated and improved concepts regarding the clinical management of chagasic individuals have emerged [11,12,13,14,15]. 

As with most infections, the process of gaining a deeper understanding of the biology of this parasite and its interaction with mammalian host cells has been accelerated through the use of experimental models in research laboratories worldwide, through both cell culture and animal models. Cell culture is an adequate model for investigating aspects of *T. cruzi*, such as its invasion capacity, intracellular replication, tropism and biochemical and immunological interactions in human/rodent cardiomyocytes; in Vero cells (a renal epithelial cells from the African green monkey); and in cervical cancer-derived cells of Henrietta Lacks (HeLa) cells [16,17,18]. Cell culture is also a primary model used to study the sensitivity of a parasite to drugs before advancing to an animal model [19]. Although cell culture models provide an environment controlled by researchers, they lack the numerous interactions observed in the complex in vivo setting. In vitro systems are also limited by their intrinsic dependency on the biological characteristics of the mammalian cells used in the experiment, including the nutritional medium, the time of infection and the temperature. Therefore, findings obtained in cell culture may be useful for understanding certain aspects of parasite biology but fail to reproduce the rich interactions observed in conditions in vivo. For example, in addition to the infection of a particular cell type, the in vivo environment promotes the interaction of the parasite with hormones, lipids, antibodies and other soluble proteins, all dependent on the genetic and/or epigenetic mammalian host background [20]. 

Rodents, especially mice, are the group of animals most frequently used to study *T. cruzi* infections. Not only are these animals simple to breed and handle in laboratory conditions, but the different lineages of mice (e.g., Swiss, C57BL/6, A/J, BALC/c, AKR, DBA) and rats (e.g., Wistar) are easily infected with various strains of *T. cruzi*. In general, experimental models in rodents are the best for studying early aspects of the interaction of *T. cruzi* with mammalian hosts, including replication in the blood and tissues, quantification of the immune response pattern, the description of intracellular and pathological responses and chemotherapy trials [21,22,23,24,25]. In these animals, there is significant parasitemia that is accompanied by anti-*T. cruzi* antibody and T cell responses [26,27,28,29,30,31]. The end of parasitemia (acute phase) is followed by the formation of fibrotic scars in muscle tissues (cardiac or skeleton muscle) as a consequence of the inflammatory process during the initial phase of the infection, which is dependent on the genetic background of the specific host and parasite strain [24,32]. It is difficult to define when the processes of “chronification” and the “chronic phase” actually start in mice. For this reason, rodents are not considered the best choice for studying chronic events in *T. cruzi* infection, particularly because of their short biological lifespan.

In this context, a few animal models have been suggested for studying the more chronic aspects of experimental *T. cruzi* infection. The dog model is currently believed to be the “gold-standard” for studying chronic *T. cruzi* infection. *T. cruzi* infection in dogs reproduces several of the pathological and clinical aspects seen in chagasic individuals, especially in the heart, including heart failure, enlargement of the cardiac chambers and electrophysiological alterations [33,34,35,36]. For reasons including the ethical issues, costs and limited genetic diversity of infectable species and parasite strains, these animals are most useful for pre-clinical therapeutic trials before advancement to humans [37,38]. 

*T. cruzi* infection in humans is significantly more complex than the diversity of presentations observed in the many strains of mice and rats and in dogs. Where transmission still occurs, humans are believed to be infected early in childhood and do not usually experience severe acute infections [11]. Indeed, the majority of individuals will never have acute symptoms (or these will be mild enough to go unnoticed), and only a minority (approximately 30%) will have a degree of organ involvement later in life [12,13,14,15]. In fact, this is the most frequent scenario in the Americas, where, despite active transmission in some places, chronic Chagas infection is the most significant presentation. Indeed, the majority of individuals are IgG-positive for chagas and have detectable parasites (via culture or PCR) in blood but never develop clinical disease, the so-called indeterminate form of Chagas disease. However, approximately 30% will develop some degree of clinical presentation later in life, usually after the first decade, and, more frequently, some degree of cardiac involvement. This is quite a significant number of chronically infected individuals who will progress to chronic Chagas disease. Persistent infection is clearly important, as individuals with no or little clinical alterations may undergo progress to disease or to more severe disease throughout life, clearly suggesting that treatment may be beneficial not only to those with indeterminate form but also to those with the milder forms of Chagas disease [39,40]. The latter clinical findings are not mirrored in the animal models of *T. cruzi* infection.

Therefore, we need to be clear when studying Chagas disease. The term chronic Chagas infection should be used to refer to those individuals with chronic infection but not any significant clinical presentation; alternatively, one should use the term indeterminate form of Chagas disease. In many studies, these patients are referred to as patients with asymptomatic Chagas disease. The latter is not a meaningful term, as an asymptomatic individual (not reporting any symptoms) may present with highly significant clinical alterations (e.g., Marked ECG changes that may not be accompanied by any symptoms or significant ventricular function that is not yet clinically apparent) and greater risks of progression and eventually death [41,42,43]. The term Chagas disease should only be used for humans with a clinical presentation of the disease and, for the reasons mentioned above, never in reference to experimental studies on rodents or even dogs. One should be extremely precise when describing the value and limitations of experiments on animals to avoid clearly undesirable extrapolations from limited animal models to the complex disease observed in humans. The title, summary and manuscript itself need to clearly indicate that it is experimental *T. cruzi* infection, and not Chagas disease, that is being studied. Moreover, experimental data involving *T. cruzi* must clearly describe the species and lineage of the laboratory animal, the discrete typing units (DTUs) and strain of the parasite, the infection dose (parasite load/animal), the period of infection, the route of the parasite inoculum and the gender of the animal. This is not to say that animal models are not useful for studying *T. cruzi* biology, providing insights into the human disease, aiding in the development of novel anti-parasites or even understanding basic immunology; however, caution needs to be used when extrapolating these findings to humans. This is true for animal models of any human disease but even more so for Chagas disease because of the variations in its clinical presentation and chronicity [11,12,13,14]. By clearly separating Chagas disease from chronic chagasic infection and from experimental *T. cruzi* infection, we hope that less confusion will be created in this field, and the value and limitations of a certain study will be more clearly perceived. Such an understanding is needed if we are to transition current knowledge towards preventive and curative therapies that will truly help chagasic individuals. 
